# Causes of Failure in the Treatment of Uterine Ulcer

**Published:** 1862-11

**Authors:** Robert Ellis

**Affiliations:** Obstetric Surgeon to the Chelsea and Belgrave Dispensary


### SELECTED.

### CAUSES OF FAILURE IN THE TREATMENT OF THE UTERINE ULCER.

By ROBERT ELLIS, ESQ.,
*Obstetric Surgeon so the Chelsea and Belgrave Dispensary.*

A considerable acquaintance with the treatment of the uterine ulcer has forced upon me the conviction that, notwithstanding all that has been done in the elucidation of this disease (or rather class of diseases), the subject is still very imperfectly understood. However unlikely this may appear, the statement admits of sufficient corroboration, and may be supported by abundant testimony. The articles which have already appeared in *The Lancet* on the varieties of the uterine ulcer were in a special manner intended by me to set this subject in a practical light, and to show the advantages of careful diagnosis as conducive to success in treatment. Because of the failures of others, and out of an inquiry into the causes of my own, those articles were prepared, and the importance of a strictly discriminative treatment was sought to be enforced. As a practical summary of the proper treatment of this or any other disease may be gathered from an investigation into the causes of a frequent ill-success, I venture to append the present to the former papers, and it is not improbable it may be found to be the most useful of the number. I will first show, by one out of many cases which have come under my notice, that there is a need for this investigation, and then enumerate the commoner causes of unsuccessful treatment.

Mrs. C. L——, aged thirty, married nine years, applied for advice under the following circumstances. She had been for three years under the care of a physician for uterine disease, and in the course of that time had been cauterized with nitrate of silver upwards of *one hundred times.* At the expiration of this period, thinking her case desperate, and having been told by her attendant that she still remained uncured, she submitted her case to me. She complained of great weakness, pain in the back, bearing down, and constant brownish discharge. She had experienced one or two bad confinements and one or two miscarriages with great hemorrhage. She was pale and worn in expression of countenance, and dejected as to her future prospects of health. Examination disclosed a large ulcer, of the character I have designated "fungous"—an ulcer, on an atheromatous basis, occupying both lips of the cervix uteri, and reaching high up the canal. What effect the large number of cauterizations she endured might have had on this ulcer I could not perceive. Possibly it might have been large formerly; but I think more probably it was a little if at all altered by the applications made to it. It had been treated by means of a small cylindrical glass speculum, and so mildly that on no single ocsasion was pain ever experienced by the patient. The cause of failure in this case in all probability lay, first in the imperfect diagnosis of the character of the ulcer, and secondly in the most inefficient and imperfect means adopted for its treatment. It was a pleasure to tell this suffering lady that in a few weeks the whole mischief would be removed and her disease cured.* It might not be easy to find a more conspicuous example of failure in treatment than that here presented, but it is by no means difficult to adduce many similar instances. Cases are constantly coming under notice which in many of their features present the parallel of this.

The principal causes of failure in treatment may be found under the following heads:

1st. *Errors of Diagnosis.*—I believe this to be one of the most constant reasons for the ill success and the slow progress of treatment in numbers of cases presented to our consideration. I have known the malignant ulcer—true cancer of the cervix—mistaken for the simple sore, and treated with escharotics, to the sad detriment of the patient. The simple ulcer has also frequently been mistaken for the cancerous, and the sufferers left unaided to struggle with a painful and depressing yet an easy curable disease. The "indolent" ulcer has been treated as the "inflamed," and the patient put to bed for five or six weeks, during which she has been repeatedly leeched. The "diphtheritic" ulcer is not so easily to be mistaken; yet it is liable to be, and has been, confounded with the "inflamed;" and it is a serious mistake for the patient if it be treated with some kinds of escharotics. The "fungous" ulcer may also (as in the case already quoted) be so trifled with—as if it were

* One or two resolute applications of the stronger caustics, followed by a thorough penetration of the canal by the lunar caustic, have verified this statement, and the patient is at this present writing nearly well.

a simple and easily curable sore—as to baffle the surgeon and wear out the patient. Of the importance of a right diagnosis in syphilitic ulcer of the cervix—a disease happily very rare —it is unnecessary to speak. There is little real difficulty in coming to a correct diagnosis as to these various forms of uterine ulcer; and if the reader will refer back to the five articles preceding this, and examine the characteristic evidence of each phase of the legitimate reward of care in diagnosis, it may be hoped that the cause of failure now treated of will be diminished in frequency as these diseases are more carefully investigated.

2nd. *Errors of Treatment.*—Of this, probably, all surgeons must be found more or less guilty. Since the instrumental treatment of uterine ulceration is of the present day, we could not but suffer for a time from the want of previous experience and a sound basis of knowledge. But these errors are no longer justifiable; and, the differing forms of uterine disease being well understood, true principles of cure may be laid down. The application of leeches to a fungous, diphtheritic, senile, indolent, malignant, or specific ulcer, is a mistake which no one ought to make—yet it is frequently made. On the other hand, the administration of wine or high-class tonics—quinine, iron, and strychnia—is a not less serious mistake, if we are treating the inflamed ulcer. The selection of an inappropriate escharotic is also an error of no little moment—the nitrate of silver to the senile, diphtheritic or malignant ulcer. In some of these cases but little harm may be done; but in nearly all, little or no good will ensue; and in one or two, positive mischief may be the result. A reliance on injections for the cure of any form of uterine ulcer is a most common and therefore a very serious error in treatment. The wife of an officer in the army has just applied to me, who for two years had strong injections of alum and sulphate of zinc administered to her daily, for an indolent ulcer of the cervix, and it has continued unaltered in spite of this treatment;—it is probably worse than it was. I have observed therefore with great regret the introduction of the system of frequent irrigation as a means of cure in these diseases. Disappointment will certainly follow a dependence on such means.

3rd. *Inefficiency of the Means Employed.*—It might seem impossible to look at the inflamed, hypertrophied, ulcerated, pussecreting structures of the diseased uterine cervix, without at the same time being impressed with the fact that no trivial agent would suffice to effect its restoration to a healthy state.

Elsewhere in the human body such an ulcer would be pretty sure to receive the firm and uncompromising attention of the surgeon. It is certainly a disease not to be cured—not, at least, soundly cured—by any agents the action of which is merely superficial. What is required is, to substitute healthy for diseased action; and the most rapid method of doing this is cauterization by the stronger escharotics. Even in the use of these a distinction is to be drawn. Potassa fusa is applicable for the melting down of a stony hypertrophy. The acid nitrate of mercury has a caustic and also an alterative action, and is applicable to certain states of the inflamed ulcer. The strong nitric acid, saturated with nitrate of silver, is fit for the treatment of the fungous ulcer, being both a powerful escharotic and an astringent. For milder cases, the nitrate of silver, very firmly applied, and allowed to lie for some seconds on the part affected, is useful. These agents have a real power over the cure of these diseases, by the side of which a medicated injection counts for but little. Yet there may be an inefficient use even of these means, powerful though they are. Neglect to remove the ropy discharges will go far to neutralize the action of any one of them—and there is no neglect more common. Neglect of applying the escharotic sufficiently high up the canal is a most frequent cause of failure. Several cases have occurred to me in which the failure of cure has its origin solely in this neglect, the disease apparently lurking high in the canal, and creeping down after the lapse of a certain time. The constant danger of fracture in using the cylinder of nitrate of silver has often led to its very inefficient employment. To obviate this I have for many years made use of an instrument in which I have passed a platinum pin, through a hollow cylinder of the caustic, and thus rendered it impossible to be broken off.† It is thus possible, were it to be desired, to pass the stick of caustic beyond the os internum, and to ensure its safe return. A too frequent cauterization is also a frequent cause of failure coming under this category, and it is particularly observable in obstetric practice at public institutions, at which the attendance of the patients is not so systematic as in private practice. The result is very frequently that the cure is always commencing and never progressing. But, in the long run, the only evil arising out of this is the great prolongation of the time of cure. A most important and common cause of inefficient treatment is to be found in

† This instrument has been well made for me by Mr. Coxer.

the use of imperfect instruments. Of the caustic-holder I have already spoken. The right form of speculum is not of less moment. I believe the failure of treatment in the case placed at the commencement of this article to be in the main traceable to the use of a cylindrical speculum exclusively. The only purpose for which this form of speculum is valuable is in the application of the fluid escharotics, which, but for its protection, are apt to run down and do mischief. It cannot be too strongly insisted upon, that a speculum, to be of real use, must be capable of opening out the lips of the cervix, as by this means alone can the canal be thoroughly cauterized.

4. *Neglect of accessory means* constitutes another very common cause of failure. Particularly, would I refer to the neglect of daily injections. The instruments formerly universally recommended for this purpose to sufferers from uterine disease, and still in very general use, are amongst the most barbarous and inefficient that can be conceived. The glass female syringe I have extracted in jagged fragments from the person of one of my patients who had recourse to it, and had broken it in the canal. The syringe of Gooch, and all others similar to it, are loathsome, and as likely to do harm as good. The clean and elegant instrument known as the uterine douche fulfils every purpose for injection, and a steady use of it (in the unimpregnated state) is a powerful aid to the cure of uterine disease. A due attention to the laws of hygiene is not less important; and, most of all, the securing an effectual state of rest—mechanical and, as far as may be, of physiological rest also. With regard to the use of medicines, it is just possible that here a little neglect may be really wholesome, for the patient has too often been put through all the formularies, and is weary of taking medicine in vain. The state of the bowels, and of the digestive functions, may not, however, be disregarded. It is, of course, perfectly probable that, notwithstanding all neglect of accessories, the patient will still be cured, if she be well managed in other respects. But this is to be remembered—that her cure will be longer, will be more painful, and is less safe than under a more perfect system.

5. *Imperfect cure* of the ulcer is a constant source of failure to the obstetric surgeon. After a few cauterizations the sore takes on such an improved look that he may think further attention unnecessary. The patient derives a certain amount of temporary good, and is satisfied; but the lapse of a year, often of less, will test the soundness of his work, and he will find to his annoyance that the whole malady has to be treated over again. The condition of the structure below the ulcer was overlooked in his estimate of the cure, and as the cauterization was not deep enough to modify that, the disease returned. Much pains, patience and time are really indispensable to the solid cure of the disease; and it is only a loss of all if the surgeon hurry his case to a premature close. It must also not be forgotten that the ulcer may be lurking high in the cervical canal, and amongst the folds of the arbor vitæ. A thorough cure will generally be a permanent cure.

The following table may prove useful as an appendage to this article, and exhibits the varieties of the ulcers of the cervix uteri in their order of frequency, with their diagnostic characters and appropriate treatment:

| VARIETIES. | CHARACTERS. | TREATMENT. |
|---|---|---|
| 1. *Indolent Ulcer.* | Cervix hypertrophied, of a pale pink and hard. Os patulous to a small extent. Ulcer of a rose-red. Granulations large, flat, insensitive, and the edge of the ulcer sharply defined. Discharge: mucous, with a little pus, and occasionally a drop of blood. | For a few times the caustic pencil. Afterwards, several applications of solution of nitrate of silver in strongest nitric acid. |
| 2. *Inflamed Ulcer.* | Cervix tender, hard, a little hypertrophied, hot and red. Vagina hot and tender. Ulcer of a vivid red. Granulations small and bleeding. A vivid red border around the ulcer. Discharge: a muco-pus, yellow and viscid, with frequently a drop of bright blood entangled in it. | Occasional leeching; hip-bath (warm); emollient injections. Then acid nitrate of mercury several times, succeeded by the solid lunar caustic potassa fusa or cum calce. |
| 3. *Fungous Ulcer.* | Cervix soft, spongy to the touch. Os wide open, so as to admit the finger. Ulcer large, pale, studded with large and friable granulations. Discharge: a glairy, brownish mucus, frequently deep tinged with blood. | At first the caustic pencil. Subsequently nit. acid solution of nitrate of silver, or acid nitrate of mercury; electric or actual cautery. |
| 4. *Senile Ulcer.* | Cervix small, red, a little hard. Ulcer small, extremely sensitive, of a bright red color. Granulations very small, extremely sensitive, of a bright red color. Discharge: a thin muco-pus. | Potassa fusa, or strong nitric acid with nitrate of silver, once or twice, at *long intervals.* Then, solid sulphate of copper in a pencil. |
| 5. *Diphtheritic Ulcer.* | Cervix of ordinary size: a little hot, dry, and tender. Ulcer covered with patches with a white membrane, adhering closely; irritable, and readily bleeding beneath. Discharge: a thin, acrid mucus without pus, but occasionally tinged with blood. | At first electric cautery, potassa cum calce, or acid nitrate of mercury, two or three times, at long intervals, *No nitrate of silver* Subsequently, stimulant applications—tincture of iodine or sulphate of copper. |

I may be allowed in conclusion, to express my conviction that the more carefully these varieties of the uterine ulcer are studied, the greater will be found the practical value of their diagnosis, and the fewer of the instances of failure in their treatment.—*London Lancet.*

The Turkish Bath in the Treatment of Insanity.—Dr. Power, resident physician of the Cork Lunatic Asylum, in a speech delivered on the occasion of the presentation of an address and testimonial to Dr. Bartes, on April 23, 1862, recommended in highly eulogistic terms a trial of the Turkish bath in the treatment of the insane. Speaking of his own experience of its effects he said: "Of course, out of more than 500 patients in the Institution all were not expected to recover, nor were they all under treatment for the purpose; but the best way of showing the effects of the bath would be by statistics. It was only fair to conclude that if the proportion of cures had been greater since the introduction of the Turkish bath than before it, this bath must have had some influence in producing that desirable result. I see by my notes that for the year ending March, 1861, the cures were 59 per cent.; but for the nine months ending 31st December last, during which period the bath has been in use, the per centage of cures was 76—that is, 74 had been cured out of 96 entered. That was more than double the number of cures produced in any asylum in England. The patients, after the first few baths, all seemed to be much pleased with it, and were always longing for the time when it was to be administered. Those who had suffered a relapse, after being sent out cured, showed no unwillingness to return to the Asylum; and even asked to be taken there at once, in order that they might get the bath, as they considered that nothing else would cure them. I have never seen any ill effects from the bath, except a little nausea and a slight fainting in a few instances, but after a bath or two those effects disappeared. Up to that time I have used it on more than 900 cases, and since March, 1861, 30 idiotic patients have been removed to a higher class, and rendered capable of enjoyment and of doing work about the establishment. I would recommend the introduction of the Turkish bath into all public Institutions, and I am firmly convinced that it has as beneficial an influence on the system as air and exercise."—*Medical Times and Gazette.*

